# Low gamma‐butyrobetaine dioxygenase (BBOX1) expression as a prognostic biomarker in patients with clear cell renal cell carcinoma: a machine learning approach

**DOI:** 10.1002/cjp2.315

**Published:** 2023-03-02

**Authors:** Kyu‐Shik Kim, Kyoung Min Moon, Kyueng‐Whan Min, Woon Yong Jung, Su‐Jin Shin, Seung Wook Lee, Mi Jung Kwon, Dong‐Hoon Kim, Sukjoong Oh, Yung‐Kyun Noh

**Affiliations:** ^1^ Department of Urology Hanyang University Guri Hospital, Hanyang University College of Medicine Guri Gyeonggi‐do Republic of Korea; ^2^ Department of Pulmonary, Allergy and Critical Care Medicine, Gangneung Asan Hospital University of Ulsan College of Medicine Gangneung Gangwon‐do Republic of Korea; ^3^ Department of Pathology, Uijeongbu Eulji Medical Center Eulji University School of Medicine Uijeongbu Gyeonggi‐do Republic of Korea; ^4^ Department of Pathology Hanyang University Guri Hospital, Hanyang University College of Medicine Guri Gyeonggi‐do Republic of Korea; ^5^ Department of Pathology, Gangnam Severance Hospital Yonsei University College of Medicine Seoul Republic of Korea; ^6^ Department of Pathology Hallym University Sacred Heart Hospital, Hallym University College of Medicine Anyang Gyeonggi‐do Republic of Korea; ^7^ Department of Pathology, Kangbuk Samsung Hospital Sungkyunkwan University School of Medicine Seoul Republic of Korea; ^8^ Department of Internal Medicine Hanyang University Hospital, Hanyang University College of Medicine Seoul Republic of Korea; ^9^ Department of Computer Science Hanyang University Seoul Republic of Korea; ^10^ School of Computational Sciences Korea Institute for Advanced Study Seoul Republic of Korea

**Keywords:** renal cell carcinoma, machine learning, low gamma‐butyrobetaine dioxygenase, CD8+ T cells

## Abstract

Gamma‐butyrobetaine dioxygenase (BBOX1) is a catalyst for the conversion of gamma‐butyrobetaine to l‐carnitine, which is detected in normal renal tubules. The purpose of this study was to analyze the prognosis, immune response, and genetic alterations associated with low BBOX1 expression in patients with clear cell renal cell carcinoma (RCC). We analyzed the relative influence of BBOX1 on survival using machine learning and investigated drugs that can inhibit renal cancer cells with low BBOX1 expression. We analyzed clinicopathologic factors, survival rates, immune profiles, and gene sets according to BBOX1 expression in a total of 857 patients with kidney cancer from the Hanyang University Hospital cohort (247 cases) and The Cancer Genome Atlas (610 cases). We employed immunohistochemical staining, gene set enrichment analysis, *in silico* cytometry, pathway network analyses, *in vitro* drug screening, and gradient boosting machines. BBOX1 expression in RCC was decreased compared with that in normal tissues. Low BBOX1 expression was associated with poor prognosis, decreased CD8+ T cells, and increased neutrophils. In gene set enrichment analyses, low BBOX1 expression was related to gene sets with oncogenic activity and a weak immune response. In pathway network analysis, BBOX1 was linked to regulation of various T cells and programmed death‐ligand 1. *In vitro* drug screening showed that midostaurin, BAY‐61‐3606, GSK690693, and linifanib inhibited the growth of RCC cells with low BBOX1 expression. Low BBOX1 expression in patients with RCC is related to short survival time and reduced CD8+ T cells; midostaurin, among other drugs, may have enhanced therapeutic effects in this context.

## Introduction

In 2019, there were 73,820 cases of renal cell carcinoma (RCC) diagnosed in the United States, with an expected death rate of 14,770. RCC accounts for 80–90% of kidney cancers, with systemic malignancy in 2–3% of cases [1]. RCC occurs in 4% of adults and, in 2018, it was the sixth most common cancer‐related cause of death worldwide [1, 2]. RCC is classified into several different histological subtypes, each with different biological characteristics: clear cell (ccRCC, 70–80% of all RCCs), papillary (10–15% of all RCCs), chromophobe (3–5% of all RCCs), and others (Xp11 translocation carcinoma, collecting duct carcinoma) [3]. Among these, ccRCC is the most common subtype of renal cancer and has high metastasis and mortality, as well as poor sensitivity to chemotherapy and radiation therapy. Surgical treatment after early detection of local RCC is the most effective therapy [3].

At the time of diagnosis, 15% of patients with RCC are diagnosed with advanced or metastatic disease [4]. Over the past decade, biomarker studies have been performed using blood rather than in tissue obtained from invasive biopsy [5]. Some of the tumor markers identified to date are as follows: survivin (BIRC5), X‐linked inhibitor of apoptosis (XIAP), myeloid cell leukemia‐1 (MCL‐1), hypoxia inducible factor/hypoxia‐induced factor (HIF1α, HIF2α), nuclear factor erythroid 2‐related factor 2 (NRF2), mouse double minute (MDM2, MDM4), p53, Kirsten rat sarcoma viral oncogene homolog (KRAS), and protein kinase B (AKT) [6]. Recent studies demonstrate that inhibitors of vascular endothelial growth factor (VEGF) and the mammalian target of rapamycin (mTOR) pathway improve the objective response rate and lead to favorable survival in metastatic renal cancer [7, 8]. Other studies have shown the efficacy of tyrosine kinase inhibitors (TKIs) and immune checkpoint inhibitors as therapeutic agents, but with no significant effect in patients with advanced and metastatic RCC [9, 10].

Gamma‐butyrobetaine dioxygenase (BBOX1) acts as a catalyst for the conversion of gamma‐butyrobetaine to l‐carnitine in the final step of the l‐carnitine biosynthesis pathway [11]. In humans, BBOX1 is found in normal kidney (high), normal liver (moderate), and normal brain (very low). Additionally, large‐scale microarray data analysis showed that BBOX1 may be related to cancer, such as of the breast, cervix, kidney, and skin [12, 13].

BBOX1 is an important gene for triple‐negative breast cancer (TNBC) tumorigenesis. BBOX1 protects the calcium channel inositol‐1,4,5‐trisphosphate receptor type 3 (IP3R3). The oncogenic role of IP3R3 is well described in multiple cancers and relies on its role in calcium release from the endoplasmic reticulum (ER) [14]. Cancer cells rewire calcium signaling to meet their growth needs [15]. The calcium signal, on the one hand, maintains mitochondrial activity for energy production; on the other hand, it supports mammalian target of rapamycin complex 1 (mTORC1)‐mediated glycolysis and other biosynthetic processes. Both mechanisms contribute to tumorigenesis and cell survival [16]. Another study showed that genetic depletion or pharmacologic inhibition of BBOX1 restricts TNBC tumor growth *in vitro* and *in vivo* [16].

The present study aimed to evaluate clinicopathological factors and survival rates according to BBOX1 expression in patients with RCC in our cohort and The Cancer Genome Atlas (TCGA) database [17]. In evaluating BBOX1 expression, our cohort used immunohistochemical analysis of protein expression, and TCGA data used RNA levels. We investigated tumor‐infiltrating immune cells and gene sets related to BBOX1 with gene set enrichment analysis (GSEA) and pathway network analysis. Through *in vitro* drug screening, we analyzed drug candidates to which RCC with low BBOX1 expression is sensitive. Moreover, we analyzed the effect of BBOX1 on the survival of patients with RCC using gradient boosting machine (GBM) learning [18].

## Materials and methods

### Patient selection

This study included 247 RCC patients who underwent surgery at Hanyang University Hospital (HYH) from June 2006 to March 2017. Of 239 cases with follow‐up data or paraffin blocks, 203 with ccRCC were selected. This study was performed according to the criteria of The Reporting Recommendations for Tumor Marker Prognostic Studies [19]. The inclusion criteria were as follows: (1) patients with microscopic features and a medical history of primary ccRCC confirmed by a pathologist and (2) patients not receiving concurrent prior neoadjuvant chemoradiotherapy. Patients with missing paraffin blocks of tumor samples or incomplete clinical outcomes were excluded. T and N stages, age, sex, histologic features, and death/recurrence/metastasis were investigated.

### Tissue microarray construction and immunohistochemistry

Tissue microarray (TMA) blocks were assembled using a tissue array instrument (AccuMax Array; ISU ABXIS Co., Ltd., Seoul, Korea). We used 3‐mm‐diameter tissue cores (tumor components in a tissue core >70%) from each donor block. Four‐micrometer sections were cut from the TMA blocks using routine techniques. Immunostaining for BBOX1 (1:100, Santa Cruz Biotechnology, Dallas, TX, USA) was performed using Bond Polymer Refine Detection System (Leica Biosystems Newcastle Ltd., Newcastle, UK) according to the manufacturer's instructions.

**Figure 1:**
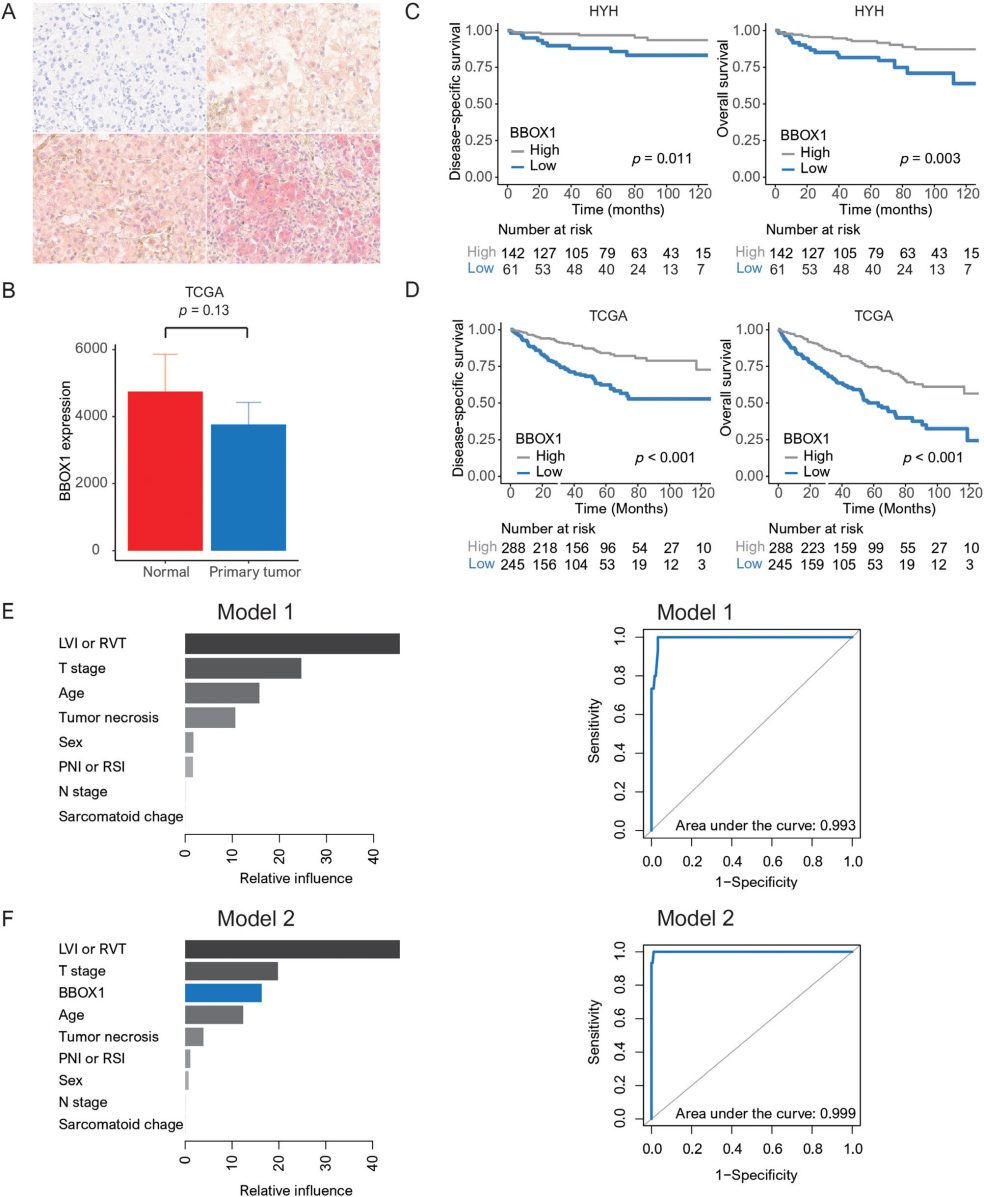
(A) The intensity of staining was scored as negative (left top), weak (right top), moderate (left bottom), or strong (right bottom) (original magnification ×200). (B) Bar plots of TCGA cohort data: *BBOX1* expression was lower in primary tumors (*p* = 0.13). (C) Survival analyses of the HYH cohort: low BBOX1 expression was associated with poor DSS and OS (*p* = 0.011 and 0.003, respectively). (D) Survival analyses of the TCGA cohort: low *BBOX1* expression was associated with worse DD and OS (all *p* < 0.001). (E and F) Supervised ML models for survival prediction using a GBM. The covariates included the confounding factors: (E) model 1; BBOX1, T stage, N stage, sex, age, histologic grade, LVI or RVT, PNI or RSI, sarcomatoid change, tumor necrosis versus (F) model 2; T stage, N stage, histologic grade, LVI or RVT, PNI or RSI, tumor necrosis. ROC curves were generated based on a multivariate Bernoulli model.

BBOX1 cytoplasmic staining intensity in tumor cells was scored on a scale of 0–3 (0 = negative; 1 = weak; 2 = moderate; 3 = strong). The percentage of BBOX1‐positive tumor cells was also scored as 1 of 4 categories: 1 (0–25%), 2 (26–50%), 3 (51–75%), or 4 (76–100%). To determine the optimal cutoff values of BBOX1 at HYH, receiver operating characteristic (ROC) curves plotting sensitivity versus 1 − specificity were used. The level of BBOX1 staining was analyzed as an immunoreactive score (IRS), which was calculated by multiplying the scores for staining intensity and proportion of positive cells. BBOX1 expression was determined to be either low (IRS < 1) or high (IRS ≥ 1) [20] (Figure 1A). The cutoff value calculated by the ROC curve was used to evaluate the relationship between cancer‐specific death events and BBOX1 expression. ROC analysis exhibited good discriminatory power for discerning death events in relation to BBOX1 expression in tumor cells (area under the ROC = 0.667).

### Gene sets, *in silico* cytometry, and network analysis based on TCGA database

We obtained 533 ccRCC cases with RNA sequence data from the TCGA database [17]. On the basis of the cancer‐specific death events in the database, *BBOX1* values were divided into low and high using the most sensitive and specific values in ROC curve analysis (cutoff value: 2,538.465). *BBOX1* expression was determined as low or high based on the cutoff value.

We analyzed significant gene sets using GSEA software (version 4.2.2) from the Broad Institute at MIT [21]. The 11,824 gene sets (hallmark [H], 50; curated [C2], 6,366; oncogenic signature [C6], 189; immunologic signature [C7], 5,219) were used to identify gene sets associated with low *BBOX1* expression. For this analysis, 1,000 permutations were used to calculate *p* values, and the permutation parameters were set to a phenotype of *p* < 0.05 and a false discovery rate of <0.35. GSEA results can determine whether there is a relationship between gene sets of H, C2, C6, and C7 and low *BBOX1* expression.

We applied CIBERSORT, known as *in silico* cytometry, to analyze the proportions of 22 subsets of immune cells using 547 genes [22]. For grouping of networks based on functionally enriched Gene Ontology (GO) terms and pathways, pathway network analyses were visualized using Cytoscape software (version 3.9.1). To interpret the molecular pathway relevance for *BBOX1*, we performed functional enrichment analysis using ClueGO software (version 2.5.8), an application for GO analysis [23, 24].

### Machine learning algorithm for validation

**Figure 2:**
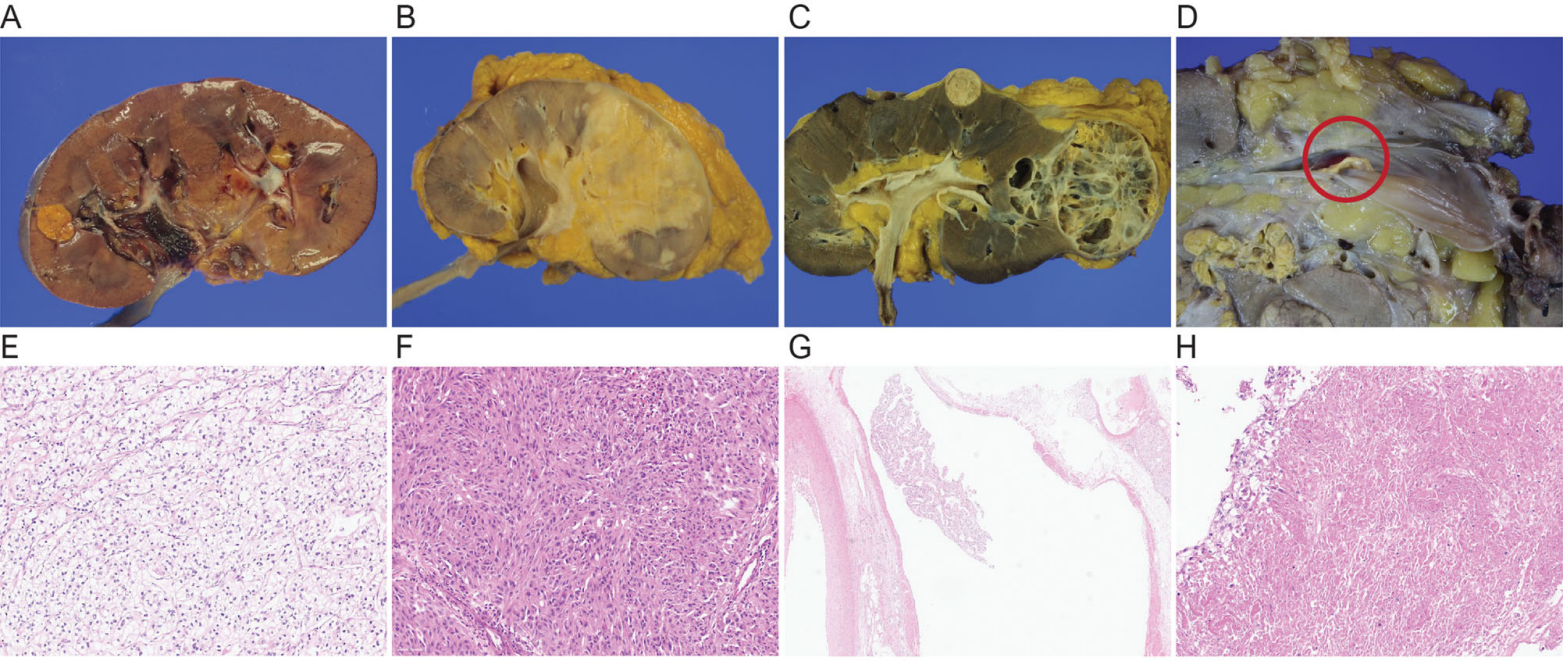
Gross view of RCC: (A) confinement to renal parenchyma, (B) RSI, (C) PFI, (D) RVT (red circle); microscopic view of RCC: (E) ccRCC, (F) sarcomatoid change, (G) vascular tumor emboli, (H) tumor necrosis.

We integrated BBOX1 protein expression with clinicopathological parameters (T stage, N stage, sex, age, histological grade known as The International Society of Urological Pathology [ISUP] grading system, lymphovascular invasion [LVI] or renal vein tumor thrombus [RVT], perinephric fat invasion [PNI] or renal sinus invasion [RSI], sarcomatoid change, tumor necrosis) into composite prognostic models for survival prediction by applying machine learning (ML) algorithms for 203 cases from the HYH cohort using IRS (staining intensity × percentage of positive cells) for BBOX1 expression (randomization: training set, 70%; validation set, 30%) (Figures 1 and 2) [25, 26]. A learning algorithm was independently applied to select and combine multiple covariates from GBM based on multivariate Bernoulli models. The hyperparameters of the ML algorithms, such as the learning rate in GBM, were optimized for each combination of selected covariates and the learning algorithm by grid search cross‐validation through a predefined range. The final optimal models were trained based on the selected covariates and optimized hyperparameters [20]. A ROC curve was used to explore the performance of the GBM method.

### Data extraction from the GDSC database

We analyzed the relationship between anticancer drug sensitivity and BBOX1 protein expression based on the Genomics of Drug Sensitivity in Cancer (GDSC) dataset [27]. Thirty ccRCC cell lines were divided into high and low groups based on the median value of BBOX1 expression. In kidney cancer cell lines with low BBOX1 expression (cell lines: TK10, LB1047‐RCC, VMRC‐RCZ, RCC‐FG2, KMRC‐1, NCC021, NCC010, U031, CAKI‐1, OS‐RC‐2, KMRC‐20, LB996‐RCC, HA7‐RCC, G‐401, RCC‐AB, SW156, RCC‐JW, and RXF393; BBOX1 < 0 based on the *z* score) or high BBOX1 expression (cell lines: CAL‐54, ACHN, BB65‐RCC, RCC‐ER, RCC‐JF, A704, LB2241‐RCC, BFTC‐909, A498, SN12C, RCC10RGB, and RCC‐MF; BBOX1 > 0), drug response was defined as the natural log of the half‐maximal inhibitory concentration (LN IC50). A drug was identified as effective when the calculated LN IC50 value was decreased in cell lines with low BBOX2 expression and increased in those with high BBOX1 expression [28, 29].

### Statistical analysis

Correlations between clinicopathological parameters and BBOX1 expression were analyzed using the *χ* ^2^ test. Student's *t*‐test and Pearson's correlation were used to examine differences among continuous variables. Disease‐specific survival (DSS) was defined as survival from the date of diagnosis to cancer‐related death. Overall survival (OS) time was defined as the time from the date of diagnosis to all‐cause death. Survival rates were compared using the log‐rank test and Cox regression analyses. A two‐tailed *p* value of ≤0.05 was considered statistically significant. All data were analyzed using R software packages and SPSS statistics (version 25.0, SPSS Inc., Chicago, IL, USA).

## Results

### Clinicopathological correlation

Low BBOX1 expression was significantly associated with high histological grade (ISUP based on conventional Fuhrman grading system: grade 1, nucleoli that are inconspicuous and basophilic at ×400 magnification; grade 2, nucleoli that are clearly visible at ×400 magnification and eosinophilic; grade 3, clearly visible nucleoli at ×100 magnification; and grade 4, extreme pleomorphism or rhabdoid and/or sarcomatoid morphology) [30] and sarcomatoid change (*p* = 0.016 and 0.019, respectively) (Table 1). In TCGA analysis, *BBOX1* expression was lower in primary tumors than in normal tissue (*p* = 0.13) (Figure 1B). Low BBOX1 expression was associated with poor DSS and OS (*p* = 0.011 and 0.003, respectively) according to both HYH and TCGA data (all *p* < 0.001) (Figure 1C,D and Table 2).

**Table 1:** Clinicopathological parameters of BBOX1 protein expression in 203 patients with clear cell RCC from the HYH cohort

Parameter	BBOX1 expression (HYH cohort)		*P* value*
	Low (*n* = 61), *n* (%)	High (*n* = 142), *n* (%)	
Age	57.4 ± 12.8	58.3 ± 12.7	0.639^†^
Sex			
Men	48 (78.7)	94 (66.2)	0.107
Women	13 (21.3)	48 (33.8)	
T stage			
1	42 (68.9)	107 (75.4)	0.431^‡^
2	3 (4.9)	6 (4.2)	
3	16 (26.2)	28 (19.7)	
4	0 (0.0)	1 (0.7)	
N stage			
0	59 (96.7)	141 (99.3)	0.448
1	2 (3.3)	1 (0.7)	
Histologic grade			
1	12 (19.7)	10 (7.0)	**0.016** ^‡^
2	26 (42.6)	77 (54.2)	
3	14 (23.0)	48 (33.8)	
4	9 (14.8)	7 (4.9)	
Lymphovascular invasion			
Absence	47 (77.0)	121 (85.2)	0.227
Presence	14 (23.0)	21 (14.8)	
Renal vein thrombus			
Absence	52 (85.2)	125 (88.0)	0.753
Presence	9 (14.8)	17 (12.0)	
Sinus fat invasion			
Absence	55 (90.2)	131 (92.3)	0.829
Presence	6 (9.8)	11 (7.7)	
Perirenal invasion			
Absence	53 (86.9)	127 (89.4)	0.776
Presence	8 (13.1)	15 (10.6)	
Necrosis			
Absence	48 (78.7)	119 (83.8)	0.5
Presence	13 (21.3)	23 (16.2)	
Sarcomatoid histology			
Absence	52 (85.2)	136 (95.8)	**0.019**
Presence	9 (14.8)	6 (4.2)	

Histologic grade, International Society of Urological Pathology grading classification based on conventional Fuhrman grading system; T or N stage, 8th edition. *p* < 0.05 is shown in bold.

* Chi‐square test.

^†^ Student's *t*‐test.

^‡^ T stage: 1 versus 2, 3, 4; histologic grade: 1 versus 2, 3, 4.

**Table 2:** DSS and OS analyses according to BBOX1 protein expression in 203 patients with ccRCC from the HYH cohort

	Univariate*	Multivariate^†^	Hazard Ratio (HR)	95% CI	
DSS					
BBOX1 (high versus low)	0.011	0.042	3.466	1.0444	11.500
T stage (1, 2 versus 3, 4)	<0.001	0.319	4.701	0.224	98.540
N stage (0 versus 1)	<0.001	0.062	4.490	0.929	21.705
Histologic grade (1, 2 versus 3, 4)	<0.001	0.197	2.984	0.567	15.700
Lymphovascular invasion (absence versus presence)	<0.001	0.257	6.195	0.265	145.068
Necrosis (absence versus presence)	<0.001	0.024	4.795	1.2333	18.654
Sex (women versus men)	0.388	0.170	2.739	0.649	11.564
OS					
BBOX1 (high versus low)	0.003	0.011	2.735	1.25555	5.961
T stage (1, 2 versus 3, 4)	<0.001	0.779	1.216	0.311	4.758
N stage (0 versus 1)	<0.001	0.027	5.125	1.20555	21.792
Histological grade (1, 2 versus 3, 4)	<0.001	0.046	2.466	1.017077	5.982
Lymphovascular invasion (absence versus presence)	<0.001	0.457	1.744	0.403	7.557
Necrosis (absence versus presence)	<0.001	0.007	3.604	1.415	9.180
Sex (women versus men)	0.259	0.138	2.082	0.790	5.488

Histologic grade, International Society of Urological Pathology grading classification based on conventional Fuhrman grading system; T or N stage, 8th edition.

* Log rank test.

^†^ Cox proportional hazard model.

We applied supervised ML models for prognostic prediction using a GBM. The covariates included confounding factors (model 1; T stage, N stage, sex, age, histological grade, LVI or RVT, PNI or RSI, sarcomatoid change, and tumor necrosis versus model 2; BBOX1, T stage, N stage, sex, age, histological grade, LVI or RVT, PNI or RSI, sarcomatoid change, and tumor necrosis with both HYH and TCGA data) and their relative importance for survival (Figure 1E,F). We found improved prognostic performance for the prediction model when BBOX1 was added (area under the curve: model 1, 0.993; model 2, 0.999).

### Gene sets and immune cell profiles

**Figure 3:**
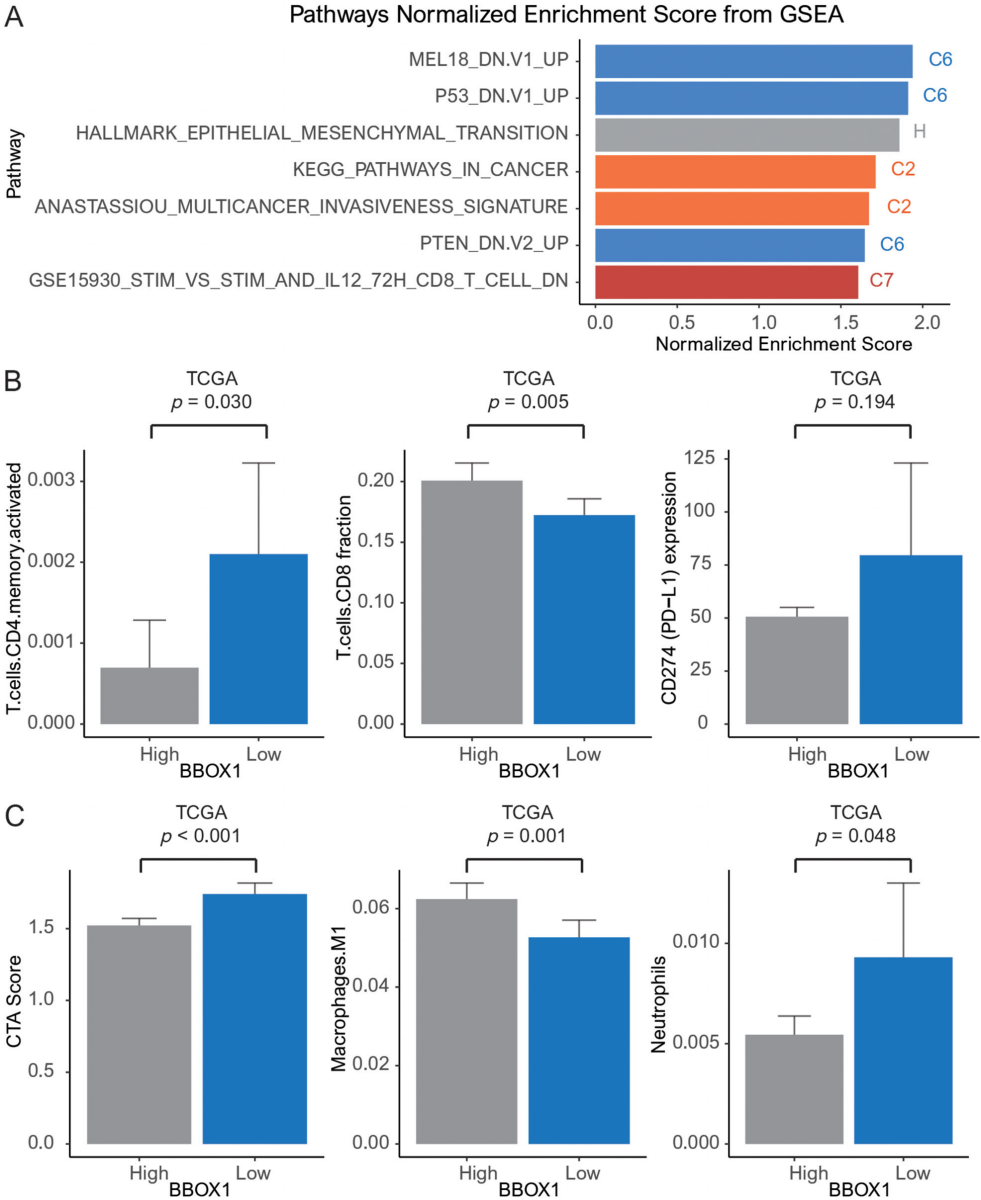
(A) Seven gene sets associated with low *BBOX1* expression: MEL18, P53, EMT, KEGG cancer pathway, invasiveness, PTEN, CD8+ T cells; (B) bar plots showing the relationship between *BBOX1* expression with the following parameters: activated memory CD4+ T cells, CD8+ T cells, CD274 (PD‐L1), CTA score, M1 macrophage, and neutrophil (*p* = 0.018, <0.001, <0.001, and 0.011, respectively) (error bars: standard errors of the mean).

We conducted GSEA to detect significant gene sets associated with low *BBOX1* expression using TCGA data. We found seven significantly enriched gene sets, such as Mel‐18, P53, epithelial mesenchymal transition (EMT), Kyoto Encyclopedia of Genes and Genomes (KEGG) cancer pathway, invasiveness signature, phosphatase and tensin homolog (PTEN), and CD8+ T‐cell downregulation (Figure 3A).

Using *in silico* cytometry based on CIBERSORT, we investigated the relationship between *BBOX1* expression and immune activity with TCGA data. CD4+ memory T cells, CD274 (programmed death‐ligand 1 [PD‐L1]) expression, cancer testis antigen (CTA) score, and neutrophils were elevated in patients with low *BBOX1* expression compared to those with high *BBOX1* expression (*p* = 0.03, 0.194, <0.001, 0.048, respectively). Low *BBOX1* expression was also associated with decreased CD8+ T cells and M1 macrophages (*p* = 0.005 and 0.001, respectively) (Figure 3B,C).

### Pathway network analysis and *in vitro* drug screening

**Figure 4:**
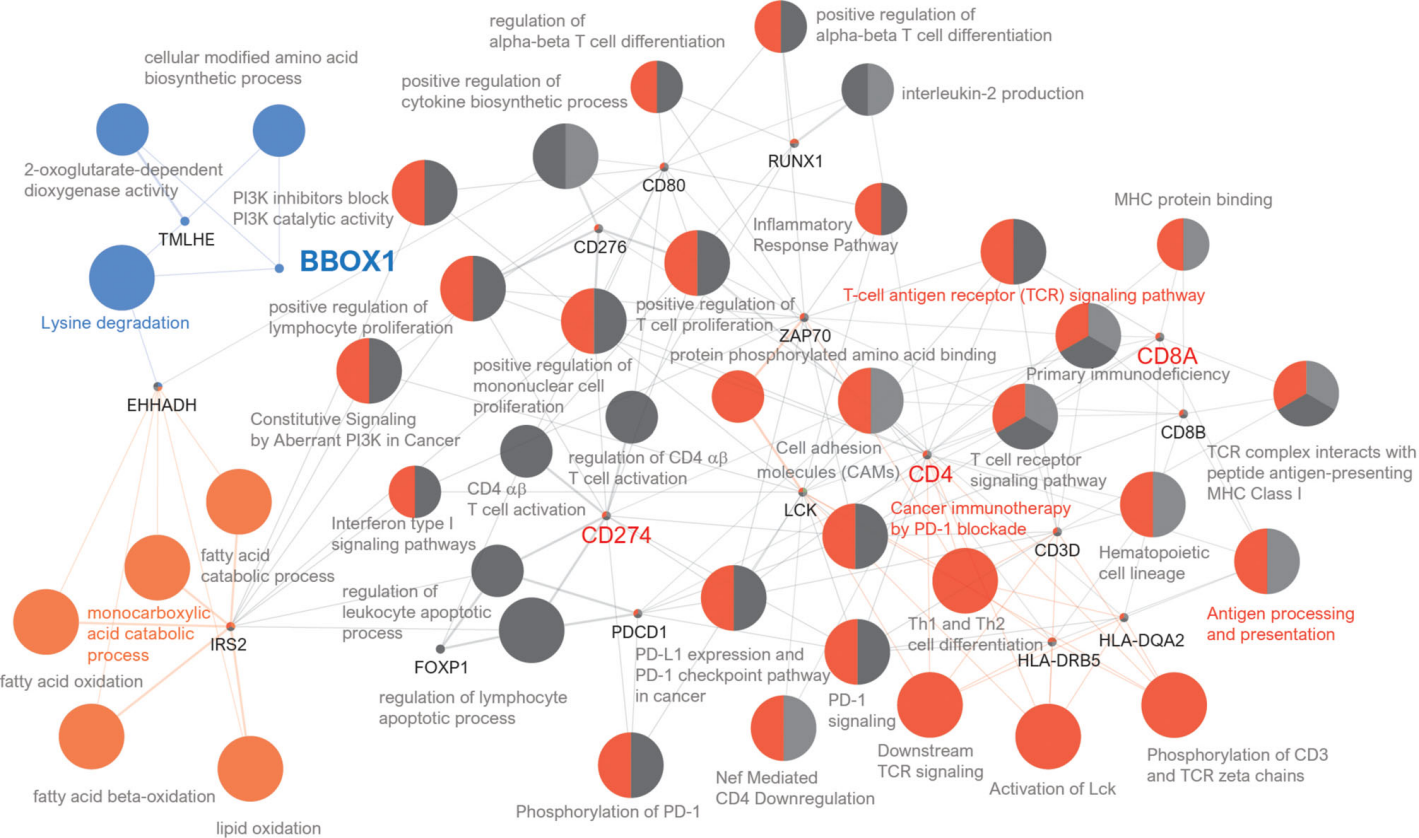
Grouping of networks based on functionally enriched GO terms and pathways related to BBOX1. The functionally grouped networks are linked to their biological functions, where only the most significantly enriched terms in the group are labeled: lysine degradation, monocarboxylic acid catabolic process, T‐cell antigen receptor signaling pathway, cancer immunotherapy by PD‐1 blockade, antigen processing and presentation as well as CD274, CD4, and CD8A.

Pathway network analysis demonstrated that BBOX1 was directly linked to lysine degradation. In contrast, BBOX1 was indirectly linked to the monocarboxylic acid catabolic process T‐cell antigen receptor signaling pathway, antigen processing and presentation, and cancer immunotherapy by PD‐1 blockade (Figure 4).

**Figure 5:**
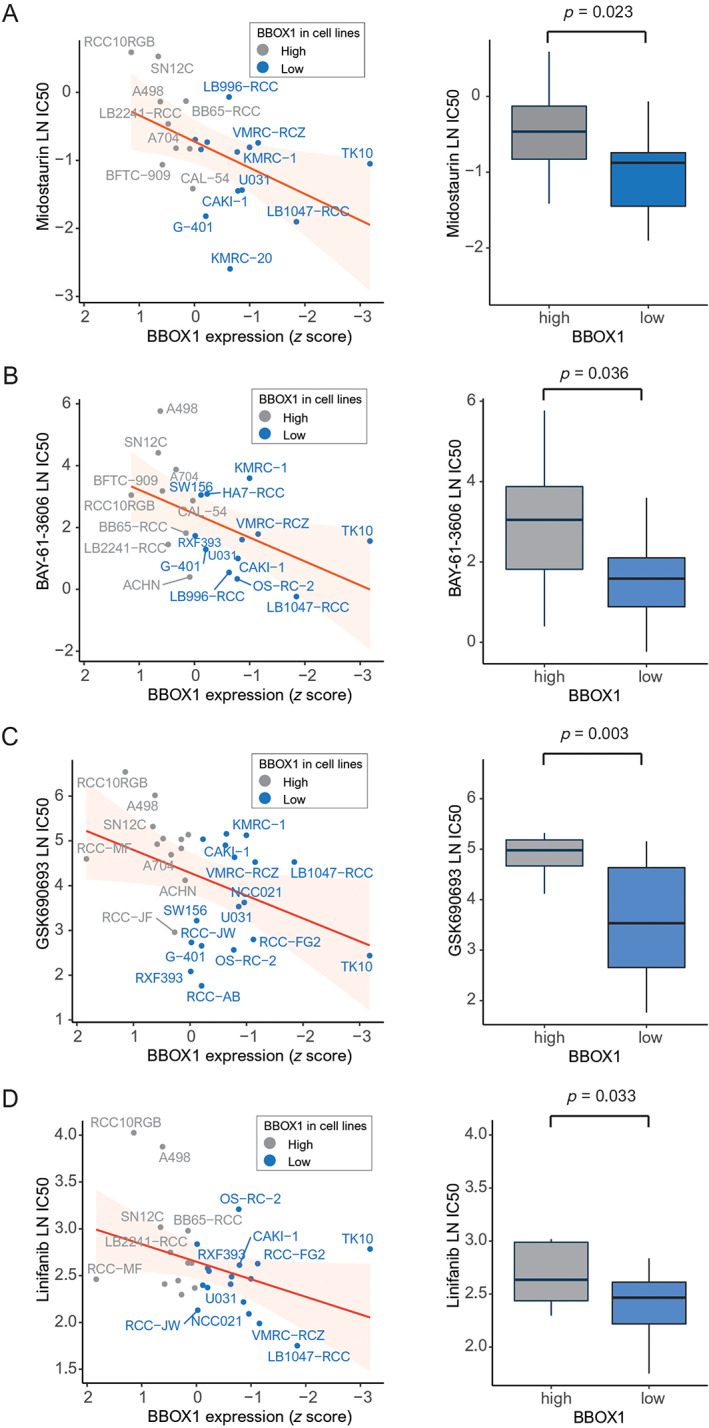
Pearson correlations (left) and box plots (right) showing the natural log LN IC50 of (A) midostaurin, (B) BAY‐61‐3606, (C), GSK690693, and (D) linifanib, the potent anticancer drugs against RCC cell lines with low BBOX1 expression.

Using GDSC datasets, including the LN IC50, we analyzed drug sensitivity in 30 RCC cell lines (Figure 5). Pearson correlation analysis showed a high positive correlation between BBOX1 and the LN IC50 value of 316 anticancer drugs. Midostaurin, BAY‐61‐3606, GSK690693, and linifanib effectively inhibited the growth of renal cancer cells with low BBOX1 expression.

## Discussion

RCC is a heterogeneous disease, making it difficult to predict clinical behavior and prepare therapeutic plans. This study demonstrates that low BBOX1 expression is associated with high histological grade and worse DSS and OS in patients with ccRCC. In TCGA, *BBOX1* expression levels were decreased in primary RCC. Low BBOX1 expression might therefore play an important role in uncovering the development and progression of ccRCC.

An important role in malignant tumors arising in the stomach [31], colon [32], cervix [33], and ovary [34] has been reported for BBOX1. A study utilizing ovarian cancer cells demonstrated that BBOX1 silencing is related to anti‐apoptotic and pro‐proliferative properties [34]. However, another study showed that high BBOX1 expression is associated with a high risk of colorectal cancer [35]. In the human protein atlas project, low BBOX1 expression correlated to unfavorable prognosis in renal cancer [36]. Our study revealed a similar result to the human protein atlas project. Thus, controversy still exists regarding the relationship between BBOX1 and clinical outcomes in various malignant tumors.

RCC is associated with rich leukocyte infiltrates, such as CD8+ T cells, CD4+ T cells and NK cells, as well as myeloid cells with characteristics of macrophages and neutrophils [37, 38]. RCC cells have a better capacity to recruit CD4+ T cells than do normal renal cells. CD4+ T cells can promote malignant cell growth by modulating transforming growth factor‐beta 1 (TGF‐β1), Y‐box‐binding protein 1 (YBX1), and hypoxia‐inducible factor 2 alpha (HIF2α) signals [39]. A high neutrophil count and tumor infiltrating neutrophils (TINs) might suppress an efficient immune response, with release of reactive oxygen species, and enhance RCC cell migration and invasion [40]. In the upregulation of estrogen receptor beta (ER‐β) caused by TIN, activated VEGF‐HIF‐2 signaling promotes the proliferation and invasion of RCC cells [41]. Our study showed decreased CD8+ T cells in RCC patients with low BBOX1 expression, suggesting low antitumor immune activity [42]. Low BBOX1 expression was found to be related to high CD274 expression, CTA score, and increased neutrophils, indicating poor prognosis. Decreased CD8+ T cells and increased CD274 expression in the context of low BBOX1 expression suggest resistance to anti‐PD‐L1 therapy. This could be considered for anticancer immunotherapy for RCC patients. In pathway network analyses, BBOX1 was linked to ‘lysine degradation’, ‘monocarboxylic acid catabolic process’, ‘T‐cell antigen receptor signaling pathway’, ‘cancer immunotherapy by PD‐1 blockades’, and ‘antigen processing and presentation’. Further experimental studies exploring BBOX1‐linked pathways are needed to investigate these relationships.

In RCC, histological nuclear grade, subtype, sarcomatoid component, LVI, and tumor necrosis are helpful for predicting survival [43, 44]. Previous studies of survival have reported different molecular biomarkers, such as carbonic anhydrase 9 mRNA (CAIX), VEGF, and insulin‐like growth factor‐1 [45, 46, 47]. These biomarkers are candidates to explore the VHL signaling pathway in ccRCC [48]. Other studies have suggested pAkt, PTEN, p27, and pS6 in the mTOR pathway as important for RCC development [49]. Our study revealed gene sets, such as Mel‐18, P53, EMT, KEGG cancer pathway, invasiveness, PTEN, and downregulation of CD8+ T cells related to low BBOX1. Mel‐18, a gene promoter regulator, might affect c‐myc, bcl‐2, cyclin D2, and Hox [50, 51], which induce tumor cell proliferation, metastasis, and angiogenesis [52, 53]. p53, a tumor suppressor, plays a pivotal role in apoptosis, genomic stability, and anti‐angiogenesis [54]. High expression of a mutated TP53 gene is associated with worse clinical outcomes in different types of malignancy, including RCC [55]. EMT increases cancer stem cell invasion to induce metastases [56]. The KEGG cancer pathway may represent cancer‐related signals in biological interpretation based on large‐scale molecular level datasets [57]. The tumor suppressor PTEN is a key component of signal transduction pathways for cell growth, proliferation, and apoptosis [58, 59].

The GDSC database has published *in vitro* drug screening data for cancer cell lines [28]. We found four drugs (midostaurin, BAY‐61‐3606, GSK690693, and linifanib) that effectively inhibited RCC cells with low BBOX1 expression. Midostaurin, known as PKC412, and benzoyl staurosporine are used to treat patients with CD135 (FMS‐like tyrosine kinase 3 receptor) mutations and are semisynthetic alkaloids derived from staurosporine [60]. Midostaurin inhibits growth or induces apoptosis in cancers, blocks angiogenesis, and sensitizes cancer cells to ionizing radiation [61]. Midostaurin was effective in RCC cell lines with low BBOX1 expression. A previous study using molecular docking analysis demonstrated that midostaurin, the best ligand for S100A8 and EGFR, inhibits downstream signaling in RCC [62]. BAY 61‐3606 is a highly selective inhibitor of Syk tyrosine kinase activity that induces cell cycle arrest and apoptosis [63]. BAY 61‐3606 suppressed the growth of RCC cell lines with low BBOX1 expression. GSK690693, as a selective protein kinase B (AKT) inhibitor, is a selective inhibitor of RCC with PTEN mutation. GSK690693 restores the sensitivity of PTEN‐deficient cancer cells to TKI‐mediated apoptosis [59]. Linifanib, as an adenosine triphosphate competitive inhibitor, has a selective effect against VEGF receptors and platelet‐derived growth factor receptor tyrosine kinases but minimal activity against unrelated receptor tyrosine kinases, cytosolic tyrosine kinases and serine/threonine kinases in patients with advanced RCC [64].

Our study had some limitations that should be acknowledged. First, this was a cross‐sectional analysis of BBOX1 expression that could not reveal continuous relationships over time, and it is difficult to make definitive conclusions. Second, an experimental study on the relationship between BBOX1 and immune cells could not be performed. Further *in vivo* studies are needed. Third, the association between BBOX1 expression and survival rate by other histological subtypes of RCC was not investigated. Fourth, we cannot assume that this model can be applied to other cohorts as we have not included validation data sets.

In summary, this study demonstrates that low BBOX1 expression is associated with tumor necrosis, high histological grade, and shorter survival time in ccRCC. In ML analyses, the model including BBOX1 showed better performance than that without BBOX1 for predicting survival. Low BBOX1 was found to be associated with decreased CD8+ T cells and high CD274 expression, suggesting resistance to anti‐PD‐L1 therapy. Drugs effective against RCC with low BBOX1 expression are presented, including midostaurin, BAY‐61‐3606, GSK690693, and linifanib. Further experimental studies of patients with ccRCC are needed to reach firmer conclusions, but these data will serve as a reference for improving the survival rate of patients through drug treatment.

## Author contributions

All authors were involved in theorising and writing the paper and had final approval of the submitted and published versions.

## Ethics approval

This study (including human participants) was approved by the Ethics Committee of Hanyang University Guri Hospital, Korea (GURI 2021‐12‐003‐002) and was conducted in accordance with the ethical standards of the Declaration of Helsinki as amended in 2008. The patient's medical records and samples from the Institutional Review Board (Institutional Review Board, Hanyang University Guri Hospital) were completely anonymized prior to access in September 2018.

## Data Availability

Public data used in this work can be aquired from the portal TCGA Research Network (https://gdc.cancer.gov/about-data/publications/pancanatlas). The raw experimental data and analysis and codes supporting the conclusions of this article will be made available by the corresponding author.
